# Co-evolution positions and rules for antigenic variants of human influenza A/H3N2 viruses

**DOI:** 10.1186/1471-2105-10-S1-S41

**Published:** 2009-01-30

**Authors:** Jhang-Wei Huang, Chwan-Chuen King, Jinn-Moon Yang

**Affiliations:** 1 Institute of Bioinformatics, National Chiao Tung University, Hsinchu, 30050, Taiwan; 2 Institute of Epidemiology, College of Public Health, National Taiwan University, Taipei, Taiwan; 3 Department of Biological Science and Technology, National Chiao Tung University, Hsinchu 30050, Taiwan

## Abstract

**Background:**

In pandemic and epidemic forms, avian and human influenza viruses often cause significant damage to human society and economics. Gradually accumulated mutations on hemagglutinin (HA) cause immunologically distinct circulating strains, which lead to the antigenic drift (named as antigenic variants). The "antigenic variants" often requires a new vaccine to be formulated before each annual epidemic. Mapping the genetic evolution to the antigenic drift of influenza viruses is an emergent issue to public health and vaccine development

**Results:**

We developed a method for identifying antigenic critical amino acid positions, rules, and co-mutated positions for antigenic variants. The information gain (IG) and the entropy are used to measure the score of an amino acid position on hemagglutinin (HA) for discriminating between antigenic variants and similar viruses. A position with high IG and entropy implied that this position is highly correlated to an antigenic drift. Nineteen positions with high IG and high genetic diversity are identified as antigenic critical positions on the HA proteins. Most of these antigenic critical positions are located on five epitopes or on the surface based on the HA structure. Based on IG values and entropies of these 19 positions on the HA, the decision tree was applied to create a rule-based model and to identify rules for predicting antigenic variants of a given two HA sequences which are often a vaccine strain and a circulating strain. The predicting accuracies of this model on two sets, which consist of a training set (181 hemagglutination inhibition (HI) assays) and an independent test set (31,878 HI assays), are 91.2% and 96.2% respectively.

**Conclusion:**

Our method is able to identify critical positions, rules, and co-mutated positions on HA for predicting the antigenic variants. The information gains and the entropies of HA positions provide insight to the antigenic drift and co-evolution positions for influenza seasons. We believe that our method is robust and is potential useful for studying influenza virus evolution and vaccine development.

## Background

Pathogenic avian and human influenza viruses often cause significant damage to human society and economics [1]. The influenza viruses are divided into subtypes based on major differences in the surface proteins hemagglutinin (HA) and neuraminidase (NA), which are the main targets for the human immune system. In circulating influenza viruses, gradually accumulated mutations on HA cause immunologically distinct strains, which lead to antigenic drift (named as antigenic variants). The antigenic drift often implies that vaccines should be updated to correspond with the dominant epidemic strains [1]. Mapping the genetic evolution to the antigenic drift of influenza viruses is one of great critical issues to public health. Many methods have been proposed to study the antigenic drift and vaccine development [2-6].

Retrospective quantitative analyses of the genetic data have revealed important insights into the evolution of influenza viruses [3,4]. In the current global influenza surveillance system, the ferret serum hemagglutinin-inhibition (HI) assay is the primary method to define the antigenic variants. Several studies [5,6] used statistical models to predict the antigenic variant of a given pair HA sequences based on these known HI assays and their respective HA sequences. Furthermore, Smith *et al*. [6] demonstrated that the antigenic evolution was more punctuated than the genetic evolution, and the genetic change sometime have disproportionately large antigenic effect. Recently, few studies discusses the relationship between evolution and co-mutated positions on influenza virus [4,7].

In this study, we proposed a method to predict the antigenic variants by identifying critical positions and rules which describe when one (e.g. circulating) strain will not be recognized by antibodies against another (e.g. vaccine) strain. Our method is also able to detect the co-mutated positions for predicting the antigenic variants. These critical positions and rules were evaluated on two datasets, which consist of 181 and 31,878 pairs. The results demonstrate that our model is able to reflect the biological meanings and achieve high prediction accuracy.

## Results

**Figure 1:**
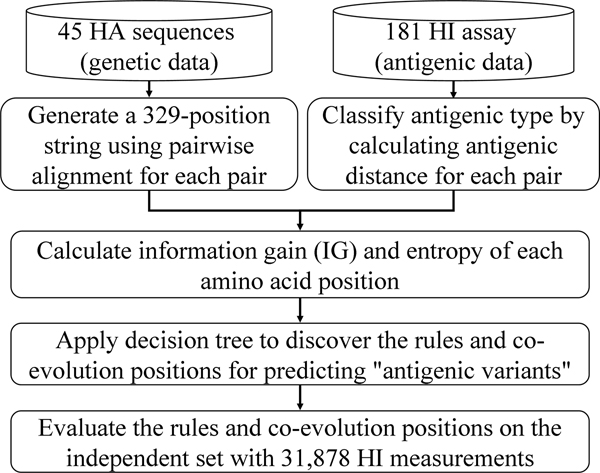
Overview of our method for predicting the antigenic variants of human influenza A/H3N2 viruses.

Figure 1 shows the overview of our method for predicting the antigenic variants of human influenza A/H3N2 virus by identifying antigenic critical positions, rules and their co-evolution on the HA.

### Data set

We collected an HI-assay data set, which contains 181 pairs of HA sequences with 45 HA (H3N2 viruses) sequences with 329 amino acids from 1971 to 2002, from related work [5]. According to this data set, we applied the decision tree C4.5 [8] to predict the antigenic variants by identifying antigenic critical positions as well as discovering the rules and co-mutated positions. In this data set, the main samples (65%, 122 pairs among 181 pairs) consist of pairs of vaccine-circulating strains, and for each pair it was known whether there is inhibition of the circulating strain by antibodies against the vaccine strain ("antigenic variants" and "similar viruses"). Vaccine strains are selected by World Health Organization (WHO) and are often the dominant strains of influenza seasons. Each pair includes the HI-assay value (i.e. antigenic distance) and a bit string with 329 binary bits by aligning a pair of HA sequences (329 amino acids). For a specific position on a pair of HA sequences, the binary value is "1 (named as mutation)" if the residue types of the two sequences on this position are different; conversely, its binary value is "0 (named as no mutation)". In general, an influenza vaccine should be updated if an HI-assay value is more than 4.0 between the current vaccine strain and the strains expected to circulate in next season [6]. The antigenic distance is defined as the reciprocal of the geometric mean of two ratios between the heterologous and homologous antibody titers [5]. Among 181 pairs of HA sequences, 125 pairs with antigenic distance ≥ 4 are considered as "antigenic variants" and 56 pairs with antigenic distance < 4 are classified as "similar viruses". For example, the antigenic distance of the pair HA sequences, A/Port_Chalmers/1/73 and A/Victoria/3/75, is 16 and this pair is considered as "antigenic variants". Conversely, the antigenic distance of the pair HA sequences, A/Wuhan/359/95 and A/Nanchang/933/95, is 1 and this pair is considered as "similar viruses".

In addition, we prepared another HI-assay data set proposed by Smith *et al. *[6] to independently evaluate our model and compare with other methods for predicting the antigenic variants. This data set consists of 253 H3N2 viruses, which are clustered into 11 antigenic groups. We assume that a virus-pair in the same antigenic group is considered as a "similar viruses" pair and a virus-pair in different groups is considered as an "antigenic variants" pair. Finally, we yielded 31,878 HI measurements and these sequences were extracted from supporting materials of publication [6].

### Antigenic critical positions on HA

**Figure 2:**
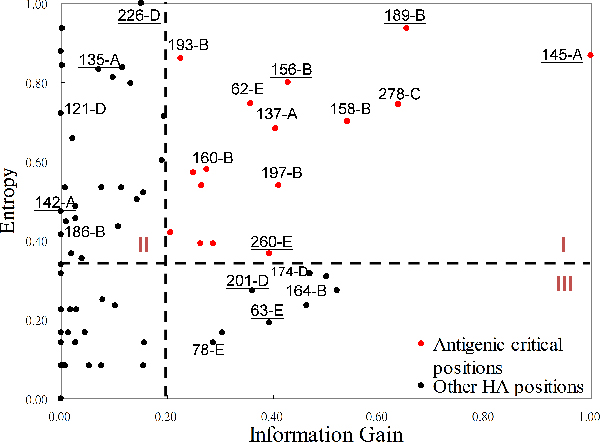
The relationship between entropies and information gains of 329 amino acids on HA protein. The positions in area I (e.g. 145-A, 189-B and 278-C) with both high entropy and high IG values are highly correlated to the antigenic variants. 145-A denotes the amino acid position 145 located at the epitope A.

In this study, we used the information gain (IG) and Shannon entropy to measure the scores of an amino acid, which locates at the specific position on HA, for discriminating antigenic variants and similar viruses (see Methods). The highest and lowest values of both IG and entropy are 1 and 0, respectively. An amino acid with high IG at a specific position implied that this position is highly correlated to the antigenic variants. An amino acid with high entropy means that this position is often mutated in the data set. Figure 2 shows the relationship of IG values and entropies of HA positions. The summaries of some amino acid positions are listed in Table 1. Of the 329 amino acids of HA, 131 positions are considered to lie in or near the five antibody combining sites (named as epitopes) which are labelled A through E [9]. The first rank (i.e. position 145-A) locates at the epitope A of HA. Its IG and entropy are 1.0 and 0.87, respectively. Among 181 pairs of HA sequences in the training set, the position 145-A mutates on 62 pairs and 61 pairs are the antigenic variants. This result implies that a mutation on this position highly induces an antigenic drift. This observation is consistent to previous results [6], that is, the single amino acid substitution N145K can be responsible for antigenic cluster transition. We observed that the other positions with high IG values obtained the similar behaviours.

**Table 1:** The entropy, information gain, and co-mutated positions of 15 amino acid positions on HA sequences

Position-epitope	Entropy	IG	Number of co-mutate positions	Co-mutated positions	Positive selection	Cluster Transition
145-A^1^	0.87	1.00	12	9,31,63,78,83,126,137,160,193,197,242,278	+ ^2^	+ ^3^
137-A	0.68	0.41	23	9,31,53,54,62,63,83,126,143,145,146,158,160,164,174,189,193,201,213,217,244,260,278		+
193-B	0.86	0.23	17	9,31,63,78,83,126,137,145,158,160,164,174,201,217,242,260,278	+	+
160-B	0.58	0.28	16	2,31,54,62,126,137,143,146,156,158,164,197,217,244,260,278		+
156-B	0.80	0.43	8	54,62,143,146,160,197,244,260	+	+

226-D	1.00	0.15	2	145,189	+	
135-A	0.83	0.07	1	165	+	
121-D	0.72	0.00	0		+	
142-A	0.47	0.00	0		+	
186-B	0.41	0.00	0		+	

164-B	0.24	0.46	6	126,137,158,174,201,217,		+
201-D	0.27	0.36	4	137,164,174,217		+
78-E	0.14	0.29	4	31,63,126,242		
174-D	0.32	0.47	4	137,164,201,217		+
63-E	0.19	0.39	6	78,83,126,137,242,278		

^1 ^The epitope of the position on HA sequence.

^2 ^the position is under positive selection defined by Bush *et al. *[3].

^3 ^the position is a cluster-difference substitution defined by Smith *et al. *[6].

**Figure 3:**
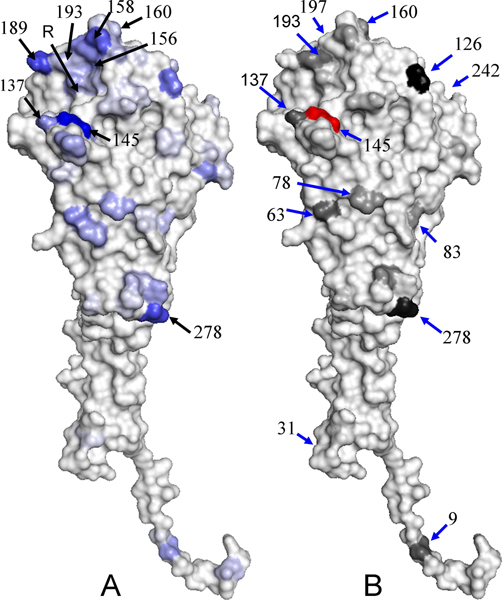
The distribution of IG values and co-mutation scores on HA structure. (A) The distribution of IG values of 329 amino acids on HA structure (PDB code 1HGF) and the R indicates the receptor binding site. The blue and gray indicate the highest IG value and the lowest IG value, respectively. (B) The structural locations and scores of 12 co-mutation positions of the position 145. These structures are presented by using PyMOL.

The relationship between IG values and entropies of 101 positions in HA is shown in Figure 2 by excluding 228 positions, which have zero for both IG and entropy. All positions can be classified into four groups according to the values of IG (antigenic degree) and entropy (i.e. genetic diversity). Those 19 positions with high IG and high entropy (i.e. Area I) are considered as critical positions in this work. According to the HA structure obtained from protein data bank (PDB code 1HGF[10]), 18 positions of them locate at five epitopes and 15 of them are on the surface (Figure 3) by using PyMOL [11]. The positions in Area II (i.e. high entropy and low antigenic degree) imply that high genetic diversity may infer low antigenic discriminating score. For example, the positions (e.g. 226-D, 135-A, 121-D, 142-A and 186-B) have high entropies and low IG values (Table 1 and Figure 2). Among 181 pairs of HA sequences, the position 226-D mutates on 61 pairs and 34 of these pairs are the antigenic variant. A low IG position indicates that a mutation on this position less preference to be an antigenic variant. Our method can avoid the disadvantage of considering only the genetic data, which was widely used in previous works.

The relationship between IG values and structural locations of 329 positions is shown in Figureure 3A. The positions with four highest IG values (i.e. 145-A, 189-B, 278-C, and 158-B) are blue and other positions are near to gray based on the IG values. The positions with high IG values are located on the protein surface. Three (145-A 189-B and 158-B) of top four IG-value positions are located around the receptor-binding site, which is key for neutralizing influenza virus. In addition, the high IG positions also prefer to locate on the top head, which are more exposed and preferable recognized by antibodies, of HA and on the interface between HA monomers.

### The rules of antigenic variants and predicting accuracies

**Figure 4:**
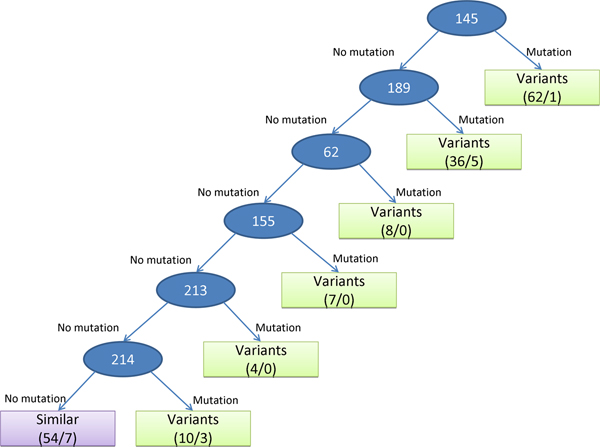
The decision tree and rules for predicting antigenic variants. Each internal node (circle) is represented as an amino acid position. The leaf node (square) includes the predicted antigenic type (i.e. "antigenic variants" and "similar viruses"), the numbers of total pairs (the first value) and predicted error pairs (the second value) by applying this rule in this node.

We used the decision tree (Figure 4) to build a model for predicting antigenic variants of human influenza A/H3N2 virus. Based on the IG values of 329 amino acid positions derived from 181 pairs in training data set, six amino acid positions are selected as internal nodes in this tree. The first rule of this tree is that the antigenic type is predicted as the antigenic variant if the position 145 is mutated, that is, the residue types of a pair of sequences on the position 145 are different. Among 181 pairs of sequences in the training set, 62 pairs can apply this rule and 61 pairs can be predicted correctly. The last rule of this tree is that the antigenic type is predicted as the similar viruses if six positions (i.e. 145, 189, 62, 155, 213, and 214) are not mutated.

**Figure 5:**
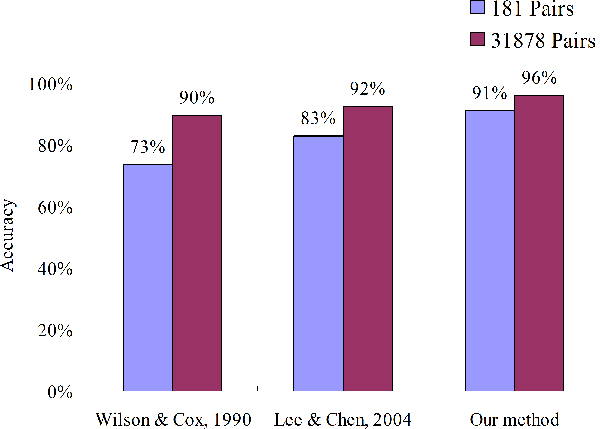
Compare our method with other two methods on predicting antigenic variants on two data sets.

Based on this model, we can derive seven rules and the predicted accuracies are 91.2% (165/181) for training data set and 96.2% (30,675/31,878) for independent data set, respectively. As shown in Figure 5 and Table 2, our method outperformed two comparative methods, i.e. Wilson & Cox (89.7%) [9] and Lee & Chen (92.4%) [5], on the independent data set. For the independent data set, the accuracies of Wilson & Cox method on predicting the antigenic variants and the similar viruses are 99.71% and 32.74%, respectively. Conversely, our model performed well for predicting the antigenic variants (99.73%) and the similar viruses (76.34%).

**Table 2:** Comparison our method with other methods for predicting the antigenic variants on 31,878 pairs

Antigenic variants	Wilson & Cox, 1990 [9]	Lee & Chen, 2004 [5]	Our method	Similar viruses	Wilson & Cox, 1990 [9]	Lee & Chen, 2004 [5]	Our method
HK68-EN72 (210 ^1^)	210	206	210	HK68 (91 ^1^)	24	52	37
EN72-VI75 (135)	135	135	135	EN72 (105)	36	79	48
VI75-TX77 (27)	27	27	27	VI75 (36)	30	36	21
TX77-BA79 (48)	48	48	45	TX77 (3)	1	2	1
BA79-SI87 (400)	400	381	400	BA79 (120)	13	46	58
SI87-BE89 (1600)	1577	863	1600	SI87 (300)	125	233	276
BE89-BE92 (3648)	3648	3648	3648	BE89 (2016)	872	1725	2016
BE92-WU95 (1596)	1542	1391	1562	BE92 (1596)	372	928	732
WU95-SY97 (448)	448	448	448	WU95 (378)	53	156	325
SY97-FU02 (96)	96	96	96	SY97 (120)	24	65	120
Other inter clusters (18890)	18889	18870	18855	FU02 (15)	15	15	15

Number of predicted pairs	27020	26113	27026	Number of predicted pairs	1565	3337	3649
Accuracy	99.71%	96.37%	99.73%	Accuracy	32.74%	69.81%	76.34%

^1 ^the number of the pairs in the cluster.

### Co-mutated positions for antigenic variants

Two amino acid positions may mutate simultaneously to cause antigenic drift or highly co-evolution in H3N2 virus. Understanding the co-mutation of amino acid position-pairs is one of the key steps to recognizing the antigen-antibody interactions. Here, we used the co-mutation score, *S*(*i*, *j*) (see Methods), between the position *i *and its co-mutated position *j *to measure the co-mutated pair (*i*, *j*) for predicting the antigenic variants. We calculated all of the co-mutated combinations (i.e. 10,100 pairs) of 101 amino acid positions which mutated more than once on 181 pairs of HA sequences in the training data set.

Table 1 show the co-mutated positions of some HA positions. In this work, the position (*j*) is considered as the co-mutation position of the position (*i*) when its co-mutation z-score (i.e. *Z*(*i*, *j*) defined as Equation (7) in Methods) is more than 2.3 because the score of the position *i *and *j *is significant (p-value is 0.01) derived from 10,100 pairs. Among 329 positions of HA sequences, 40 positions have co-mutated positions. The number of co-mutated positions for a position ranges from 0 to 23 and the total number of the significant pairs are 308 among 10,100 pairs.

In the tree model (Figure 4), the position 145-A is selected as first node and has 12 significant co-mutated positions (Table 1 and Figure 3B). The top three significant co-mutated positions of 145-A are (145-A, 126-A), (145-A, 278-C) and (145-A, 137-A). The 145-A, 278-C, and 137-A are the residues to cause the transition from the cluster EN72 into the cluster VI75 [6]. In addition, the residue 156-B has 8 significant co-mutated positions (Table 1). Seven (except position 260-E) of these 8 positions co-mutate with 156-B to cause the transition from the cluster TX77 into the cluster BK79 [6].

Table 3 shows the numbers of significant co-mutation positions on six blocks, including five epitopes and the other area on the HA protein. The numbers (24 and 19 pairs, respectively) of the co-mutation pairs, which located at epitopes A and B, are significantly higher than other block. This result implies that the mutation on epitopes A and B could yield a high probability to cause the antigenic drift. In addition, residues in epitopes A and B form 82 and 71, respectively, significant co-mutation pairs which are much higher than other blocks. On the other hand, the number (i.e. 18 pairs) of significant co-mutation pairs formed by the residues in non-epitope block is the smallest among 36 combinations of six blocks (Table 3). These results demonstrate that epitopes A and B are more important than other blocks and the five epitopes are more important than the other area. Previous works shows that epitopes A and B are more antigenic important since they are around the receptor binding site [9].

**Table 3:** The numbers of co-mutation positions of five epitopes and the other area on HA protein

	Epitope A	Epitope B	Epitope C	Epitope D	Epitope E	Other area	sum
Epitope A	15	**24**	8	11	16	8	**82**
Epitope B	**19**	15	6	13	13	5	**71**
Epitope C	**15**	11	3	5	9	4	47
Epitope D	12	**13**	3	8	6	4	46
Epitope E	**13**	11	4	6	7	3	44
Other area	4	2	1	3	4	4	18

**Figure 6:**
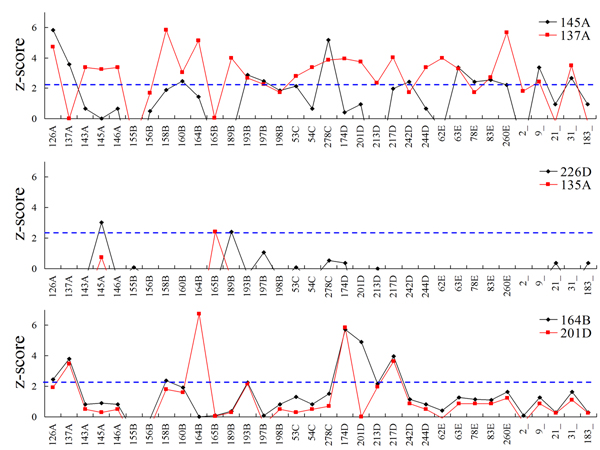
The co-mutation z-score distributions of six positions on the HA sequence. A position is considered as a co-evolution residue if its z-score is more than 2.3 (i.e. the blue line).

Figure 6 shows the distributions of co-mutation z-scores of six HA positions. The positions (i.e. 145-A and 137-A located in Area I in Figure 2) which have high IG values and high entropies, own 12 and 23 co-mutated positions (Figure 6A and Table 1), respectively. On the other hand, Figure 6B shows two positions (i.e. 226-D and 135-A located in Area II in Figure 2), which have low IG values and high entropies, own 2 and 1 co-mutated positions (Table 1), respectively. Finally, the positions 164-B and 201-D have similar distributions (Figure 6C) and their correlation coefficient is 0.73. To consider both IG values and entropies provide insight to the antigenic draft and co-evolution positions on influenza virus. These results show our method is able to identify co-mutated positions that participate in the antigenic drift for influenza seasons. These significant co-mutated positions show biological meaning.

## Discussion

Previous works using genetic data for identifying great diversity positions, that under immune selection, have shown fruitful results [3]. However, Smith *et al. *[6] demonstrated that antigenic evolution is more punctuated than genetic evolution, which implies only genetic data may not enough to detect antigenic critical positions. For example, the antigenic discriminating score (i.e. IG = 0.15) of the position 226-D is low, while its genetic diversity (i.e. entropy is 1.0) is largest. The position 226-D is also selected as positive selection [3]. According to 181 pairs in the training set, the position 226-D has 61 mutations, but 27 of them are "similar viruses" pairs. Therefore, its antigenic discriminating score is low and a mutation on this position does not cause the antigenic drift. The position 121-D, which is under positive selection, has the similar behaviour.

Although the HI assay can successfully detect antigenic drift, this assay is labour-intensive and time-consuming. Therefore, the quantity of HI data is far fewer than sequence data and sometimes have the problem of bias sampling [12]. The position 164-B, which identified by Smith *et al. *as cluster-difference substitutions from 253 sequences [6], has 28 mutations in 181 pairs and all of them happened in "antigenic variants" pairs. Mutations on this position (i.e. IG is 0.46) have high preference to antigenic variant. But our method didn't select this position because the genetic diversity (i.e. entropy is 0.24) of this position is not high enough.

In the independent data set (31,878 pairs), the accuracies of three methods are more than 96% for the "antigenic variants", but their accuracies on the "similar viruses" pairs are significantly different (Table 2). The method proposed by Wilson and Cox [9] falsely predicts 67% of "similar viruses" pairs, which implies this method is very sensitive in the same antigenic group. Compare our model with the hamming distance (HD) model based on epitope positions proposed by Lee & Chen [5], our model have high accuracies in three groups, i.e. BE89, WU95 and SY97 (Table 2). For example, for 2016 "similar viruses" pairs in the BE89 group, the HD model falsely predicted 291 pairs, which are correctly predicted by our model, and the average HD of these 291 pairs is 7.3. Most of these 291 pairs mutate on seven positions (i.e. 50-D, 80-E, 137-A, 159-B, 167-D, 173-D and 197-B). Except the positions 137-A and 197-B, the other five positions have low antigenic discriminating scores based on our model.

For each the position-pairs (*i*, *j*) and (*j*, *i*) in Figure 6, their z-scores are different because the position *i *and *j *have different antigenic discriminating scores. For example, the z-scores of position-pairs (133-A, 156-B) and (156-B, 133-A) are 5.03 and -1.13, respectively. In addition, the IG values of the positions 156-B and 133-A are 0.43 and 0.11, respectively. The antigenic effect of only mutation on the position 133-A is not significant. On the other hand, the antigenic discriminating score is significant when the position 133-A co-mutates with the position 156-B. Among 181 pairs in the training set, the position 133-A occurs 38 mutations and 32 of them are "antigenic variants" pairs, and 31 pairs of them co-mutate with the position 156-B. This position pair is observed to cause the transitions from the cluster TX77 into the cluster BK79 and from the cluster BE89 into the cluster BE92 [6].

Among 329 positions of HA sequences, 137-A, 193-B, and 160-B are top three positions with the highest numbers of co-mutated positions. The position 137-A has 23 co-mutation positions and top three pairs are (137-A, 158-B), (137-A, 260-E) and (137A, 164-B). These four positions are observed to cause the transitions from the cluster EN72 into the cluster VI75 and from the cluster VI75 into the cluster TX77 [6].

There are total 308 significant position-pairs but only 142 pairs of them observed in cluster-difference substitutions [6]. For example, 15 pairs with top 50 z-scores but not identified as cluster-difference substitution are: (83-E, 126-A), (145-A, 126-A), (193-B, 126-A), (126-A, 63-E), (278-C, 126-A), (63-E, 126-A), (137-A, 126-A) (83-E, 278-C), (193-B, 63-E), (31,9), (83-E, 63-E), (126-A, 278-C), (9,31), (275-C, 145-A) and (126-A, 145-A). Nine pairs of them could be observed in the 1976 fixation [4] in which they analyzed large amount of HA protein sequences (2248 sequences from 1968 to 2005). These results imply our method is able to detect potential co-mutated positions related to antigenic drift from limited HI-data.

## Conclusion

This study demonstrates our model is robustness and feasibility by considering both genetic and antigenic data. Based on decision tree, our method is able to identify critical amino acid positions of HA and the rules of antigenic variants for influenza H3N2 viruses. The accuracies of our method are 91.2% and 96.2% for training set and independent data set, respectively, and our method is significantly better than other two methods on these two sets. The identified critical amino acid positions are similar to related works and the co-mutated positions are able to reflect the biological meanings. We believe that our method is useful for vaccine development and the evolution of human influenza A viruses.

## Methods

### Identify antigenic critical positions on HA

In this study, positions with both highly antigenic discriminating score and highly genetic diversity are considered as antigenic critical positions. We first evaluate the genetic diversity, which commonly believed related to immune selection [3], of each amino acid position on HA. Here, Shannon entropy was used to measure the genetic diversity of an amino acid position *i *(*i *= 1~329) with 20 amino acid types and is defined as

(1) $$H(i)=-\sum_{T=1}^{20}P(A_{i}=T)\log(P(A_{i}=T))$$

where *P*(*A*_*i *_= *T*) is the probability of the position *i *with amino acid type *T*. The information gain [8] measures the score of an amino acid position on HA for discriminating between antigenic variants and similar viruses. An amino acid with high IG at a specific position implied that a mutation on this position is highly correlated to antigenic variants. The IG of the position *i *associates to antigenic type *Y *(i.e. antigenic variants (V) and similar viruses (S)) is defined as

(2) *IG*(*i*, *Y*) = *H*(*Y*) - *H*(*Y*|*i*)

H(Y) is the entropy of antigenic type *Y *and is defined as

(3) $$H(Y)=-\sum_{T\in \{V,S\}}P(Y=T)\log(P(Y=T))$$

*H*(*Y*|*i*) is the conditional entropy of *Y *when given the position *i*. Two states of the position *i *are mutation (*M*) and non-mutation (*N*). *H*(*Y*|*i*) is defined as

(4) $$H(Y|i)=-\sum_{K\in \{M,N\}}P(A_{i}=K)H(Y|A_{i}=K)$$

*P*(*A*_*i *_= * K*) is the probability of the position *i *in state *K*. *H*(*Y*|*A*_*i *_= * K*) is the entropy of antigenic type *Y *when given the position *i *in state *K*. *H*(*Y*|*A*_*i *_= * K*) is given as

(5) $$H(Y|A_{i}=K)=-\sum_{T\in \{V,S\}}P(Y=T|A_{i}=K)\log(P(Y=T|A_{i}=K))$$

For example, for the position 145, the numbers of the "mutation" and "non-mutation" are 62 and 119, respectively, among 181 pair-wise HA sequences in the training data set. For 62 mutation pairs, the numbers of "antigenic variants" and "similar viruses" are 61 and 1, respectively. The numbers of "antigenic variants" and "similar viruses" are 55 and 64, respectively, for 119 non-mutation pairs. According to these data, we can calculated that *P*(*A*_145 _= * M*) is 0.34 and *H*(*Y*|*A*_145 _= * M*) is 0.12 for the mutation state; *P*(*A*_145 _= * N*) is 0.66 and *H*(*Y*|*A*_145 _= * N*) is 1.0 for the non-mutation state. Finally, we obtained *H*(*Y*|*i*) = 0.70. The values of information gain and entropy of 329 HA positions are normalized in the range from 0 to 1.

### Discover the rules of antigenic variants

After identifying antigenic critical positions, we discovered the rules for predicting antigenic variants by applying the decision tree C4.5 [8]. These antigenic amino acid positions are considered as the attributors (features). An amino acid position with high IG was selected as an internal node in the tree to discriminate "antigenic variants" and "similar viruses" (Figure 4). According to the selected positions and constructed tree, we can easily identify the rules according to the paths from the root to the leaves of the tree.

### Predicting antigenic variants

In order to evaluate and compare our model with other methods [5,9] for predicting antigenic variants, we collected two data sets. The first data set consists of 181 pair-wise HI measurements and the second independent data set contains 31,878 HI measurements proposed by Smith *et al. *[6]. Wilson & Cox [9] suggested that a drift viral variant of epidemiologic importance usually contains more than 4 residues changes located on at least 2 of the five epitopes on the HA protein. Lee & Chen [5] proposed a model based on the hamming distance (HD) of 131 positions on five epitopes of HA to predict antigenic variants. Their model predicted a pair of HA sequences as the antigenic variants if there are more than 6 amino acid mutations between this pair HA sequences.

### Identify co-mutated positions for antigenic variants

Here, we used the decision tree hierarchy to identify co-mutation of two amino acid positions. In order to identify all potential co-mutated pairs on HA, the positions (i.e. 101 positions among 329 positions), which occur mutations in 181 pairs of HA sequences, are sequentially selected to identify its co-mutated positions. Based on these 101 positions, the total number of two-position combinations is 10,100. For each amino acid positions (*i*), the co-mutation score (*S*(*i*, *j*)) between the position *i *and its partner position *j *is defined as

(6) *S*(*i*, *j*) = *IG*_*W*_(*j*, *Y*) - *IG*_*Ri*_(*j*, *Y*)

where *IG*_*W*_(*j*, *Y*) is the IG value, which is derived from the whole data set (i.e. 181 pairs of HA sequences in the training set) using Equation (2), of the position *j*; *IG*_*Ri*_(*j*, *Y*) is the IG value of the position *j *derived from the data set R by removing the pairs, in which the position *i *is mutated, from the whole data set. The z-score of the *S*(*i*, *j*) of a pair of co-mutated positions is derived from 10,100 pairs and it is defined as

(7) $$Z(i,j)=\frac{S(i,j)-\mu }{\sigma }$$

*μ *and *σ *are the mean and standard deviation of all 10,100 position pairs. For example, position 145 (IG is 1.0) is selected as the first node in the tree. Among 181 pairs, 62 pairs are mutated on the position 145. The amino acid positions are considered as co-mutated positions of the position 145 if their IG values significantly decrease after these 62 pairs are removed from the data set. For example, the z-score of the *S*(145, 137) of the pair-positions 145 and 137 is 3.58

## Competing interests

The authors declare that they have no competing interests.

## Authors' contributions

Conceived and designed the experiments: JWH and JMY. Performed the experiments and analyzed the data: JWH and JMY. Contributed reagents/materials/analysis tools: JWH, CCK, and JMY. Wrote the paper: JWH and JMY
